# Assessment of incidence of cerebral vascular diseases and prediction of stroke risk in chronic obstructive pulmonary disease patients using multimodal biomarkers

**DOI:** 10.1111/crj.13587

**Published:** 2023-01-25

**Authors:** Marwa Y. Badr, Amira A. Elkholy, Sara M. Shoeib, Marwa G. Bahey, Esraa A. Mohamed, Alaa M. Reda

**Affiliations:** ^1^ Neurology unit, Neuropsychiatry Department, Faculty of Medicine Tanta University Egypt; ^2^ Pulmonology Department, Faculty of Medicine Tanta University Egypt; ^3^ Clinical Pathology Department, Faculty of Medicine Tanta University Egypt; ^4^ Medical Microbiology and immunology Department, Faculty of Medicine Tanta University Egypt; ^5^ Diagnostic Radiology Department, Faculty of Medicine Tanta University Egypt

**Keywords:** biomarkers, brain imaging, cerebrovascular disease, COPD, duplex, laboratory investigations, stroke risk

## Abstract

**Background:**

Early assessment of cerebrovascular disease in chronic obstructive pulmonary disease (COPD) patients is an important issue for a favorable influence on the quality of life.

**Methodology:**

This cross‐sectional case–control study was conducted on 38 eligible COPD patients (mean age 55.5 ± 11.5, 25 males, and 13 females) and 26 age‐/sex‐matched healthy controls. All participants were subjected to stroke risk screening instruments that included the Stroke Riskometer™, the Framingham 10‐Year Risk Score, the stroke risk screening tool (the Department of Disease Control of Thailand), the My Risk Stroke Calculator, and Q Stroke. Radiologically, diffusion tensor imaging (DTI) and echo‐gradient MRI (T2 star) T2 star imaging were done. Color‐coded duplex sonography was done. Laboratory investigations included C‐reactive protein (CRP), serum amyloid A, plasma fibrinogen level, serum IL6, 8‐Isoprostane, vWF and urinary albumin creatinine ratio.

**Results:**

Stroke risk screening instruments revealed a significant increase in COPD patients. DTI showed a significant bilateral reduction in fractional isotropy and a significant bilateral increase in mean diffusivity of white matter through many areas in COPD patients. Patients also had a significant increase of intima–media thickness, presence of atherosclerotic focal thicknesses or plaques on duplex sonography. There was a significant elevation of CRP, serum amyloid A, plasma fibrinogen level, serum IL6, 8‐isoprostane, von Willebrand factor (vWF), and urinary albumin creatinine ratio in COPD patients.

**Conclusion:**

COPD patients had an increased risk for stroke that could be assessed on stroke risk screening instruments, DTI, T2 star, duplex sonography, and laboratory investigation and could be correlated with the severity of the disease.

## Graphical abstract

Chronic obstructive pulmonary disease (COPD) nowadays represents a major health problem worldwide, not only for patients but also for the community, so proper prevention and management of COPD and its exacerbation are now a vital target for a better quality of life. Systemic inflammation, oxidative stress, and shared risk factors involved in COPD pathogenesis should be targeted to prevent serious comorbidities such as cerebrovascular events, so, early screening of COPD patients with elevated inflammatory markers, oxidative burden, and poor lung functions for stroke risk becomes mandatory and valuable for both patients and the community.

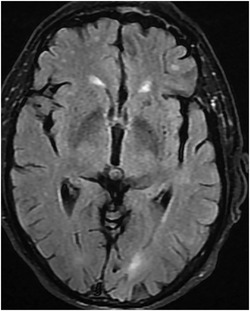

## 1 INTRODUCTION

Chronic obstructive pulmonary disease (COPD) is now considered one of the three main leading causes of death worldwide, about 3 million people died of COPD in 2012. Indoor and outdoor pollutants can cause COPD in people who do not smoke. Despite COPD being a preventable and treatable disease, it represents a major health problem due to its complications and systemic manifestations.^1^

Previous studies have reported that patients with COPD no exacerbations (COPDne) or COPDe are at increased risk of stroke. However, the risk of stroke for patients with COPDe is not completely understood. COPD is associated with chronic inflammation, oxidative stress, and increased inflammatory cells not only in airways and lung parenchyma but also in systemic circulation, which may play a role in associated comorbid conditions.^2^, ^3^

Risk stratification of stroke is clinically useful because it can help inform patients of the risk of stroke, allowing them to improve their lifestyle or make an informed medical decision. Therefore, a series of biomarkers reflecting inflammation, hemostasis, thrombosis, endothelial function, or neurohormonal activity have been evaluated as potential tools in an effort to improve risk prediction of future stroke and thereby avert future events.^4^, ^5^, ^6^

There has been an increasing emphasis on the identification of markers that represent even earlier stages of vascular brain disease. In this respect, white matter microstructure, as assessed with diffusion tensor imaging (DTI), has received increasing interest.^7^, ^8^

Intracranial atherosclerosis is a significant risk factor for ischemic strokes and transient ischemic attacks (TIAs), accounting for about 10% of such events and can be assessed by transcranial Doppler (TCD) ultrasonography. The physiologic data obtained from TCD are complementary to structural data obtained from various modes of currently available vascular imaging.^9^, ^10^

## 2 AIM OF THE WORK

This research was conducted to assess incidence of cerebral vascular diseases and predict risk for stroke in patients with COPD, using potential biomarkers including laboratory, imaging (DTI), and TCD for possible, early recognition and prevention.

## 3 SUBJECTS AND METHODS

### 3.1 Subjects

This cross‐sectional case–control study was conducted on 38 COPD patients attending inpatients and outpatients' clinics of the chest and neuropsychiatry departments, Tanta university hospitals in the period from December 1, 2021 to the end of May 2022. A total of 26 healthy control subjects who matched the patient's age, sex, and educational level were also included for analysis and comparison.

The study protocol was approved by the local institutional ethics committee of the faculty of medicine (Approval No: 35031/11/21), Tanta University, and informed consent was obtained from each participant before enrollment.

#### 3.1.1 Inclusion criteria

This included the following: (1) participants (patients and controls) aged above 40 years old and (2) COPD patients diagnosed depending upon grading of chronic obstructive pulmonary disease refined (ABCD) assessment tool and Global Initiative for Chronic Obstructive Lung Disease (GOLD) criteria.^11^

#### 3.1.2 Exclusion criteria

(1) Participants with other concomitant lung diseases. (2) Patients with previous stroke, dementia, other neurologic diseases, major head trauma, or severe organic illness. (3) Participants with atrial fibrillation, cardiomyopathy, and heart failure. (4) Patients with vasculitides. (5) Failure to comply with research protocol. (6) The presence of any contraindication for undergoing MRI scanning (e.g., claustrophobia and pacemaker).

### 3.2 Methods

Patients were subjected to history taking including age, smoking index, medical comorbidities, and complete clinical examination (general, local chest, and neurological examinations).

#### 3.2.1 Assessment of COPD status

All participants were evaluated using refined ABCD assessment tool (based on severity of symptoms assessment using modified Medical Research Council Dyspnea Scale, mMRC; COPD Assessment Test, CAT**™** score, Arabic version; and assessment of risk of exacerbations) and the GOLD criteria (based on spirometrically confirmed diagnosis via post‐bronchodilator, post‐BD, and forced expiratory volume in 1 second, FEV1/FVC < 0.7), and then assessment of airflow limitation grade based on FEV1% was predicted.^11^ These assessments were carried out by a qualified pulmonology consultant.

#### 3.2.2 Risk stratification of stroke

All patients and control subjects were subjected to risk stratification of stroke using five existing stroke risk screening tools including (1) the Stroke Riskometer™, (2) the Framingham 10‐Year Risk Score, (3) the stroke risk screening tool (the Department of Disease Control of Thailand), (4) the My Risk Stroke Calculator, and (5) Q Stroke, which were included and identified.^12^, ^13^, ^14^, ^15^ These tools were carried out by a qualified neuropsychiatric consultant.

#### 3.2.3 Radiological evaluation

All subjects were submitted to brain imaging that included white matter changes and diffusion MRI using DTI and echo‐gradient MRI (T2 star).^7^, ^8^

MR imaging was done at 1.5 T (GE Signa Explorer) by a standard eight‐channel head coil. The slice thickness was 4 mm, the matrix was 256 × 256, and the field of view was 220–240 mm. Axial 3D DTI was achieved using a single‐shot spin‐echo echo‐planar imaging (TR/TE = 7400/60 ms, 56 slices, acquisition matrix of 112 × 112 with pixel size of 2 × 2 × 2 mm^3^, interpolated to 1 × 1 × 2 mm^3^, 60 diffusion gradient directions with b = 1000 s/mm^2^, and three repeats of b = 0).^16^ Post‐processing was performed using MRI workstation software (ADW 4.7 Vantage, GE Medical Systems) by GE Software devised for tractography. The analysis was based on the evaluation of DTI parameters; fractional anisotropy (FA), mean diffusivity (MD), and relative apparent diffusion coefficient values were assessed by placing small regions of interest (ROIs) measuring 2 × 2 mm^3^ in different white matter areas in both cerebral hemispheres. All values were normalized to the values from the white matter of controlled volunteers to obtain relative values of all parameters.^16^, ^17^

T2*‐based imaging includes making gradient echo (GRE) sequences more sensitive to T2* decay by changing user‐selectable parameters such as echo time (TE), flip angle, and repetition time (TR) in an appropriate way. With these T2*‐weighted sequences, the lesion, structures, or areas of dephasing are shown as dark areas, leading to their detection or characterization.^18^

#### 3.2.4 Color‐coded duplex ultrasonography evaluation

All subjects were subjected to evaluation of extra‐ and intracranial carotid and vertebral vessels using color‐coded duplex ultrasonography. It is a noninvasive maneuver that depends on using a pulsed Doppler system with low transmitter frequency. This method allows blood flow velocities to be recorded from basal cerebral arteries through the intact skull by using transtemporal, suboccipital, and transorbital windows. The Doppler signal obtained is assigned to a specific artery based on indirect parameters: the depth of the sample volume, the position of the transducer, and the flow direction.^9^, ^10^

The extracranial carotid system was assessed by a linear array transducer of multifrequency (3–12) MHZ, real time, sagittal, coronal, and axial views, with measurement of intima–media thickness (IMT) average, peak systolic velocity (PSV), end diastolic velocity (EDV), and detection of the presence of focal thickness or plaque in either common carotid artery (CCA), internal carotid artery (ICA), or external carotid artery (ECA). Intracranial vessels were assessed by a phased array transducer of multifrequency (1–3) MHZ, transaxial, mesencephalic view through a temporal window using a Philips Ultrasound, Bothel, WA 98021 USA device.^9^, ^10^

#### 3.2.5 Laboratory investigations

Subjects were also submitted to laboratory investigations that included the following:

1. Routine laboratory test results were obtained from the laboratory reports and serum levels of C‐reactive protein (CRP), serum amyloid A, plasma fibrinogen level, and urinary albumin creatinine ratio (UACR).^5^, ^19^
2. Specific laboratory investigations:

- Blood samples: 5 ml of venous blood was drawn under complete aseptic conditions into plain tube and sodium citrated tube and then centrifuged at 3000 ×*g* for 15 min. Separated serum and plasma samples were aliquoted and stored at −20°C until used.
- The serum was used for:

1. Measurement of serum level of 8‐isoprostane using 8‐isoprostane ELISA kit from Eagle Biosciences (catalog number: 8IS39‐K01).^20^
2. Measurement of serum IL‐6 using enzyme‐linked immunosorbent assay kit for human IL‐6 from Elabscience (catalog no: E‐EL‐H6156).^19^

- The plasma was used for:

1. Measurement of plasma levels of vWF:Ag using human von Willebrand factor antigen (vWF:Ag) ELISA kit from MyBioSource (catalog number: MBS733078).^5^

### 3.3 Statistical analysis

Statistical analysis was carried out using the SPSS software statistical computer package V17. The range and mean ± standard deviation (SD) were measured for quantitative data. For qualitative data, comparison between two or more groups was conducted by chi‐square test **(**X^2^). Correlation analysis was performed by Pearson's correlation and Spearman's rho correlation tests. Significance was adopted at *p* < 0.05 for interpretation of results.^21^

## 4 RESULTS

This study included 38 COPD patients (mean age 55.5 ± 11.5 years, 25 males (65.79%), 13 females (34.21%)) and 26 controls (mean age 55 ± 10 years, 17 males (65.38%), and 9 females(34.62%)). Patients had a mean smoking index of 47.5 ± 32.5 pack/year, 20 (52.63%) of them were smokers, 8 (21.05%) of them were ex‐smokers, and the remaining 10 (26.32%) were nonsmokers (%). Controls had a mean smoking index of 27.5 ± 12.5 pack/year, 7 (26.92%) of them were smokers, 3 (11.54%) of them were ex‐smokers, and the remaining 16 (61.54%) were nonsmokers. Among patient groups, 19 (50%) had comorbidities hypertension (18), diabetes mellitus (7), chronic kidney disease (3), dyslipidemia (2), and ischaemic heart disease (1). Besides, these comorbidities showed nonsignificant positive correlation with Stroke Riskometer™ (5‐year stroke risk %), Stroke Riskometer™ (10‐year stroke risk %), Framingham 10‐Year Risk Score (%), My Risk Stroke Calculator, and QRISK®3 10‐year risk calculator (%) (*p* value 0.233, 0.270, 0.157, 0.603, and 0.157, respectively, for previous variables).

### 4.1 Assessment of COPD status results

Assessment of COPD severity in patients using refined ABCD assessment tool and the GOLD criteria showed a significant reduction of post‐BD FEV1/FVC ratio and FEV1 (% predicted) level in COPD patients than in controls with *p* value <0.001 for each. There was a significant increase of mMRC, CAT score, and risk of exacerbations in COPD patients than in controls with *p* value <0.001 for each. According to ABCD assessment tool grades, 2 (5.26%) COPD patients were classified as Group A, 9 (23.68%) COPD patients were classified as Group B, 14 (36.84%) COPD patients were classified as Group C, and 13 (34.21%) COPD patients were classified as Group D. According to the GOLD criteria, 1 (2.63%) COPD patient was classified as Group 1, 7 (18.42%) COPD patients were classified as Group 2, 22 (57.89%) COPD patients were classified as Group 3, and 8 (21.05%) COPD patients were classified as Group 4.

### 4.2 Risk stratification of stroke results

The study showed a significant increase of each Stroke Riskometer™ (5‐year stroke risk), Stroke Riskometer™ (10‐year stroke risk), Framingham 10‐Year Risk Score, My Risk Stroke Calculator, and QRISK®3 10‐year risk calculator in COPD patients than in controls with *p* value <0.001 for each. According to the stroke risk screening tool, 4 (10.53%) patients were classified as having low risk, 14 (36.84%) patients were classified as having cautious risk, and 20 (52.63%) patients were classified as having high risk, with statistically highly significant differences (*p* < 0.001) than their matched controls. Stroke Riskometer™ (5‐year stroke risk), Stroke Riskometer™ (10‐year stroke risk), Framingham 10‐Year Risk Score, My Risk Stroke Calculator, and QRISK®3 10‐year risk calculator were all directly correlated with mMRC, CAT score, risk of exacerbations, ABCD assessment tool grades, and the GOLD criteria but inversely correlated with spirometric data, post‐BD FEV1/FVC ratio and FEV1 (% predicted) level (see Supporting Information and Tables 4 and 5).

### 4.3 Radiological results

**FIGURE 1:**
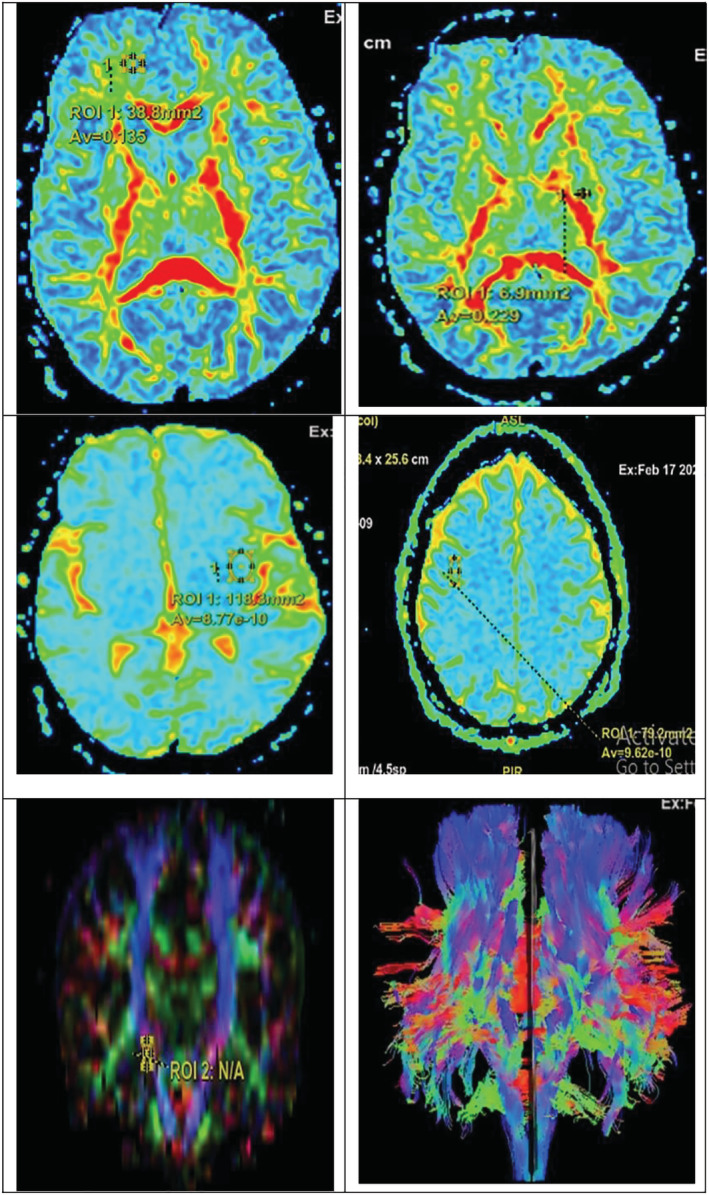
Diffusion tensor imaging (DTI) study in chronic obstructive pulmonary disease (COPD) patients with upper panel showing reduced FA in the following from RT to LT side: RT frontal subcortical tract and LT internal capsule; middle panel showing reduced apparent diffusion coefficient (ADC) in the following from RT to LT side: LT temporal lobe and RT parietal lobe; and lower panel showing positioning of the region of interest (ROI) used to measure FA and mean diffusivity (MD) including RT to LT side: RT corticospinal tract and whole tracts

DTI showed a significant reduction in fractional isotropy with a significant increase in MD in many areas including the superior occipitofrontal fasciculus (SOFF), inferior occipitofrontal fasciculus (IOFF), arcuate fibers, corpus callosum, frontal subcortical tract, parietal subcortical tract, temporal subcortical tract, cingulum, corona radiata, internal capsule, cerebral peduncle, and corticospinal tract in both hemispheres in COPD patients than in controls (see Supporting Information, Table 1, and Figure 1).

**TABLE 1:** DTI and T2 star in COPD patients and control subjects

DTI			Groups		T‐test	
			Patient	Control	t	*p* value
SOFF (FA)	RT	Range	0.19–0.44	0.31–0.44	−4.152	<0.001*
		Mean ± SD	0.299 ± 0.076	0.366 ± 0.034		
	LT	Range	0.2–0.45	0.34–0.45	−4.121	<0.001*
		Mean ± SD	0.304 ± 0.080	0.372 ± 0.033		
SOFF (MD)	RT	Range	4.6–7.4	4.66–5.2	4.332	<0.001*
		Mean ± SD	5.720 ± 1.025	4.842 ± 0.137		
	LT	Range	6.63–9	6.63–7	4.339	<0.001*
		Mean ± SD	7.521 ± 0.779	6.851 ± 0.118		
IOFF (FA)	RT	Range	0.16–0.38	0.3–0.38	−6.475	<0.001*
		Mean ± SD	0.268 ± 0.064	0.353 ± 0.022		
	LT	Range	0.17–0.39	0.32–0.38	−5.823	<0.001*
		Mean ± SD	0.276 ± 0.063	0.349 ± 0.017		
IOFF (MD)	RT	Range	7.44–10	7.44–7.91	4.177	<0.001*
		Mean ± SD	8.342 ± 0.798	7.681 ± 0.127		
	LT	Range	4.46–7.3	4.55–4.99	4.202	<0.001*
		Mean ± SD	5.652 ± 1.009	4.812 ± 0.153		
Arcuate F (FA)	RT	Range	0.2–0.43	0.33–0.41	−2.255	0.028*
		Mean ± SD	0.358 ± 0.054	0.383 ± 0.018		
	LT	Range	0.21–0.44	0.36–0.42	−2.601	0.012*
		Mean ± SD	0.363 ± 0.051	0.390 ± 0.015		
Arcuate F (MD)	RT	Range	4.68–7.4	4.77–4.97	1.174	0.245
		Mean ± SD	4.994 ± 0.538	4.869 ± 0.063		
	LT	Range	4.18–6.8	4.19–4.3	3.100	0.003*
		Mean ± SD	4.603 ± 0.583	4.247 ± 0.029		
CC (FA)		Range	0.4–0.64	0.56–0.63	−4.000	<0.001*
		Mean ± SD	0.524 ± 0.072	0.582 ± 0.018		
CC (MD)		Range	3.37–6	3.38–3.48	3.109	0.003*
		Mean ± SD	3.866 ± 0.679	3.451 ± 0.027		
Frontal subcortical tract (FA)	RT	Range	0.07–0.3	0.2–0.29	−7.300	<0.001*
		Mean ± SD	0.156 ± 0.060	0.248 ± 0.028		
	LT	Range	0.05–0.25	0.15–0.24	−4.810	<0.001*
		Mean ± SD	0.122 ± 0.055	0.180 ± 0.032		
Frontal subcortical tract (MD)	RT	Range	5.11–8	5.11–5.25	7.520	<0.001*
		Mean ± SD	6.461 ± 0.861	5.187 ± 0.040		
	LT	Range	4.53–7	4.55–4.7	8.079	<0.001*
		Mean ± SD	5.798 ± 0.729	4.638 ± 0.049		
Parietal subcortical tract (FA)	RT	Range	0.06–0.28	0.2–0.28	−5.987	<0.001*
		Mean ± SD	0.149 ± 0.060	0.223 ± 0.025		
	LT	Range	0.03–0.23	0.15–0.21	−5.219	<0.001*
		Mean ± SD	0.131 ± 0.057	0.190 ± 0.015		
Parietal subcortical tract (MD)	RT	Range	5.01–7.5	5.03–5.15	7.711	<0.001*
		Mean ± SD	6.373 ± 0.831	5.112 ± 0.032		
	LT	Range	4.87–7.56	4.87–5	8.704	<0.001*
		Mean ± SD	6.387 ± 0.854	4.925 ± 0.044		
Temporal subcortical tract (FA)	RT	Range	0.05–0.29	0.19–0.28	−7.003	<0.001*
		Mean ± SD	0.149 ± 0.059	0.235 ± 0.026		
	LT	Range	0.01–0.19	0.12–0.17	−3.561	0.001*
		Mean ± SD	0.104 ± 0.044	0.136 ± 0.017		
Temporal subcortical tract (MD)	RT	Range	3.84–6.5	3.85–3.99	11.018	<0.001*
		Mean ± SD	5.135 ± 0.563	3.912 ± 0.045		
	LT	Range	7.69–10	7.7–7.81	6.299	<0.001*
		Mean ± SD	8.592 ± 0.674	7.757 ± 0.036		
Cingulum (FA)	RT	Range	0.24–0.48	0.37–0.47	−7.521	<0.001*
		Mean ± SD	0.301 ± 0.066	0.405 ± 0.030		
	Lt	Range	0.36–0.66	0.56–0.65	−8.583	<0.001*
		Mean ± SD	0.438 ± 0.087	0.590 ± 0.025		
Cingulum (MD)	RT	Range	2.93–5.5	2.93–3.04	9.147	<0.001*
		Mean ± SD	4.065 ± 0.591	3.001 ± 0.034		
	LT	Range	3.5–6	3.51–3.66	6.011	<0.001*
		Mean ± SD	4.533 ± 0.808	3.577 ± 0.040		
Corona radiata (FA)	RT	Range	0.14–0.4	0.31–0.39	−7.053	<0.001*
		Mean ± SD	0.274 ± 0.055	0.355 ± 0.024		
	LT	Range	0.28–0.53	0.42–0.51	−6.935	<0.001*
		Mean ± SD	0.382 ± 0.058	0.467 ± 0.026		
Corona radiata (MD)	RT	Range	5.01–7.3	5.04–5.16	9.798	<0.001*
		Mean ± SD	6.039 ± 0.485	5.103 ± 0.032		
	LT	Range	5.61–8	5.62–5.77	6.232	<0.001*
		Mean ± SD	6.507 ± 0.643	5.718 ± 0.045		
Internal capsule (FA)	RT	Range	0.25–0.5	0.4–0.49	−6.406	<0.001*
		Mean ± SD	0.349 ± 0.070	0.442 ± 0.027		
	LT	Range	0.25–0.52	0.42–0.51	−6.768	<0.001*
		Mean ± SD	0.354 ± 0.079	0.463 ± 0.027		
Internal capsule (MD)	RT	Range	4.92–7.41	4.91–5.1	9.267	<0.001*
		Mean ± SD	6.079 ± 0.599	4.985 ± 0.054		
	LT	Range	4.35–7	4.37–4.55	4.068	<0.001*
		Mean ± SD	5.105 ± 0.788	4.474 ± 0.046		
Cerebral peduncle (FA)	RT	Range	0.49–0.75	0.65–0.74	−6.590	<0.001*
		Mean ± SD	0.600 ± 0.064	0.687 ± 0.026		
	LT	Range	0.34–47	0.51–0.6	0.775	0.441
		Mean ± SD	1.696 ± 7.548	0.545 ± 0.030		
Cerebral peduncle (MD)	RT	Range	4.44–7	4.45–4.6	6.627	<0.001*
		Mean ± SD	5.457 ± 0.711	4.529 ± 0.049		
	LT	Range	7.05–9.4	7.06–7.22	4.614	<0.001*
		Mean ± SD	7.718 ± 0.630	7.146 ± 0.044		
Corticospinal tract (FA)	RT	Range	0.24–0.51	0.41–0.51	−7.958	<0.001*
		Mean ± SD	0.342 ± 0.068	0.455 ± 0.031		
	LT	Range	0.28–0.55	0.39–0.55	−6.817	<0.001*
		Mean ± SD	0.363 ± 0.083	0.480 ± 0.034		
Corticospinal tract (MD)	RT	Range	4.03–7.6	4.05–4.22	7.262	<0.001*
		Mean ±SD	5.187 ± 0.731	4.143 ± 0.049		
	LT	Range	5.71–8	5.71–5.89	7.789	<0.001*
		Mean ± SD	6.818 ± 0.660	5.805 ± 0.049		

Chi‐square		*N*	%	*N*	%	X^2^	*p* value
T2 star	Normal	18	47.37	21	80.77	7.235	0.007*
	Microbleeds	20	52.63	5	19.23		

Abbreviations: CC, corpus callosum; COPD, chronic obstructive pulmonary disease; DTI, diffusion tensor imaging; FA, fractional anisotropy; IOFF, inferior occipitofrontal fasciculus; LT, left; MD, mean diffusivity; RT, right; SOFF, superior occipitofrontal fasciculus.

* Statistically significant p < 0.05.

**FIGURE 2:**
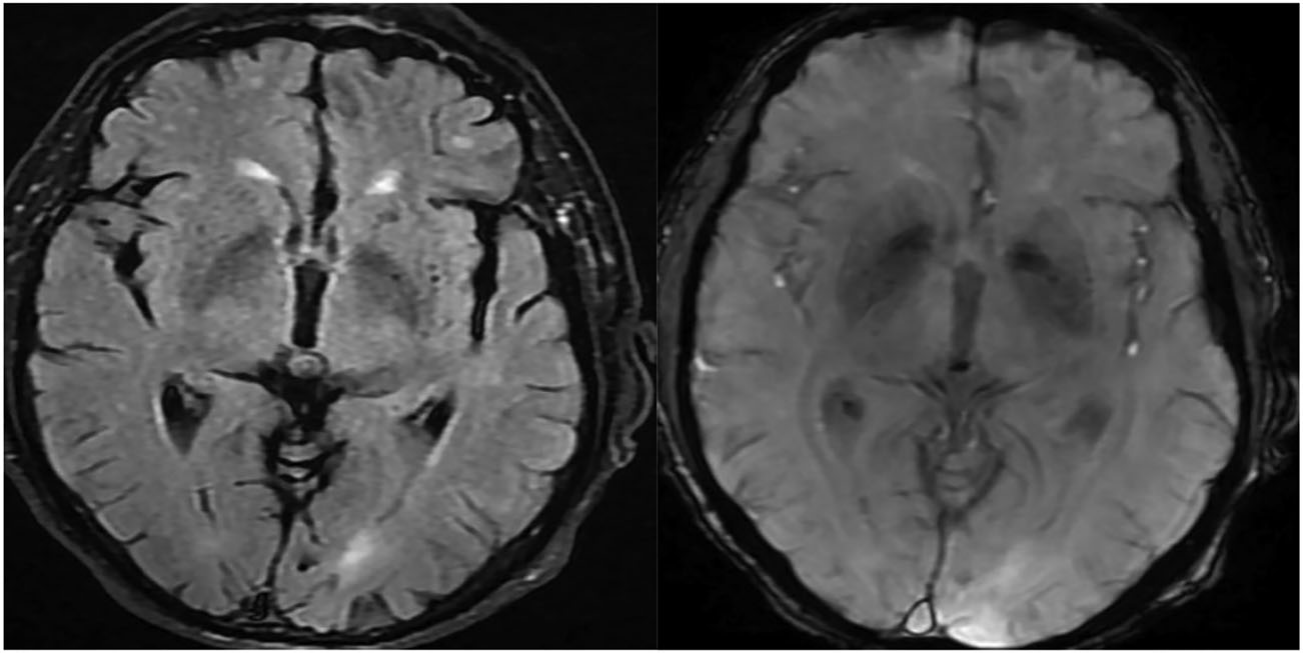
MRI study in chronic obstructive pulmonary disease (COPD) patients with tiny low signal foci of hemosiderin of microbleeds in the FLAIR film RT image and T2 star in LT image

T2 star revealed either normal findings in 18 (47.37%) COPD patients versus in 21 (80.77%) controls or microbleeds in 20 (52.63%) COPD patients versus in 5 (19.23%) controls with statistically significant differences for both previous variables with *p* value 0.007 (see Supporting Information, Table 1, and Figure 2).

MD of the SOFF, IOFF, arcuate fibers, corpus callosum, frontal subcortical tract, parietal subcortical tract, temporal subcortical tract, cingulum, corona radiata, internal capsule, cerebral peduncle, and corticospinal tract in both hemispheres was all directly correlated with mMRC, CAT score, risk of exacerbations, ABCD assessment tool grades, and the GOLD criteria but inversely correlated with FEV1/FVC ratio and FEV1 level.

FA of the SOFF, IOFF, arcuate fibers, corpus callosum, frontal subcortical tract, parietal subcortical tract, temporal subcortical tract, cingulum, corona radiata, internal capsule, cerebral peduncle, and corticospinal tract in both hemispheres was all directly correlated with FEV1/FVC ratio and FEV1 level but inversely correlated with mMRC, CAT score, risk of exacerbations, ABCD assessment tool grades, and the GOLD criteria.

### 4.4 Color‐coded duplex ultrasonography results

**FIGURE 3:**
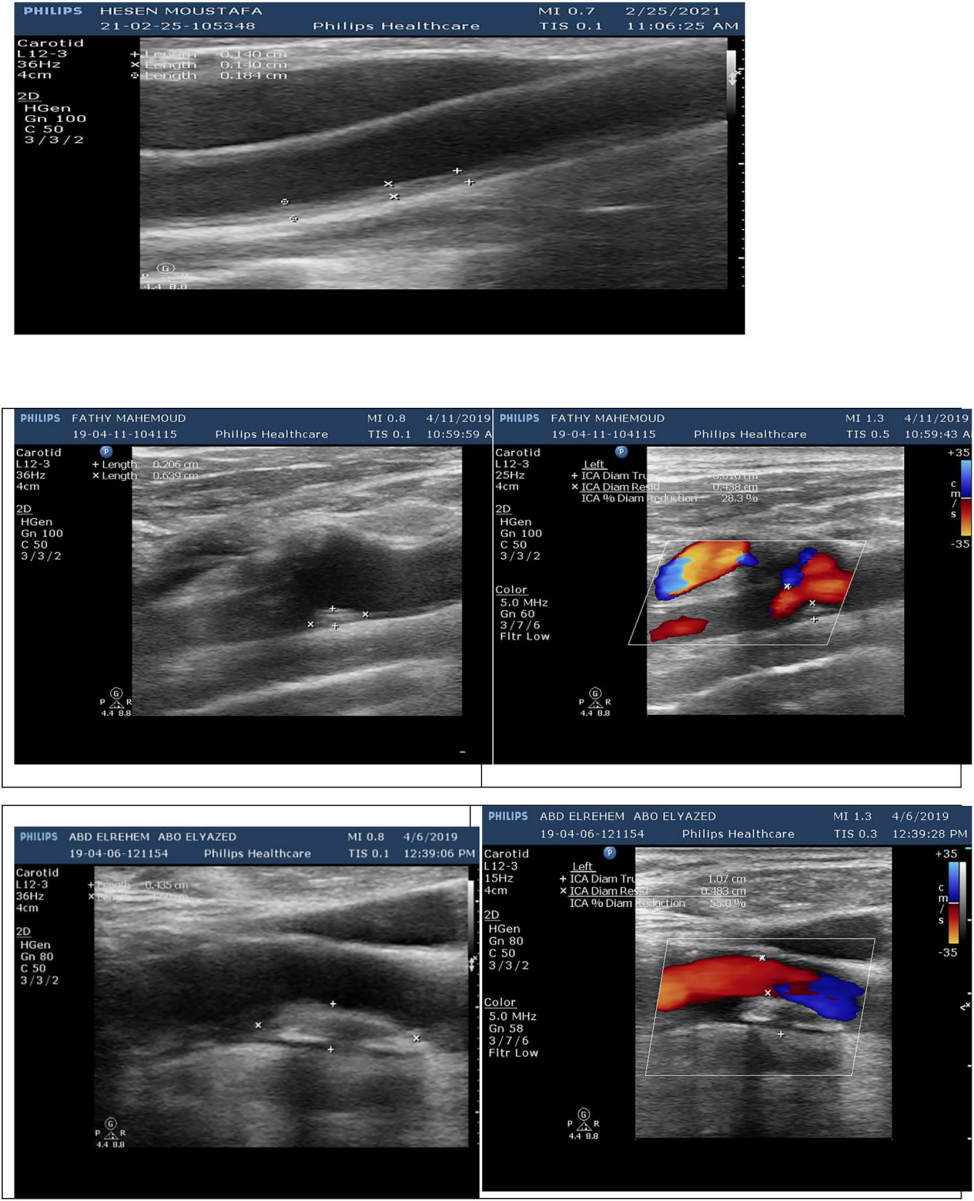
Duplex ultrasonography study in chronic obstructive pulmonary disease (COPD) patients with upper panel showing increased intima–media thickness (IMT) (about 0.15 cm); the second panel showing homogenous hyperechoic focal thickness in the distal common carotid artery (CCA) measuring 0.36 cm × 0.20 cm, making diameter stenosis 28.3%; and the third panel showing homogenous hyperechoic plaque in the internal carotid artery (ICA) measuring 1.00 cm × 0.54 cm, making diameter stenosis 55%

Color‐coded duplex ultrasonography showed a significant increase of IMT in COPD patients than in controls (*p* value <0.001). Focal thicknesses were present in 15 (39.47%) COPD patients with statistically significant differences than their matched controls with *p* value 0.038, whereas plaques were present in nine (23.68%) COPD patients with statistically significant differences than their matched controls with *p* value 0.032. Meanwhile, there were no statistically significant differences regarding location, echogenicity, thickness, and luminal diameter stenosis of either focal thickness or plaque in COPD patients than in controls (Table 2) (Figure 3). IMT, thicknesses of both focal thicknesses and plaques, and luminal diameter stenosis of both focal thicknesses and plaques were positively correlated with mMRC, CAT score, risk of exacerbations, ABCD assessment tool grades, and the GOLD criteria but negatively correlated with FEV1/FVC ratio and FEV1 level (see Supporting Information and Tables 4 and 5).

**TABLE 2:** Duplex findings in COPD patients and control subjects

Duplex findings		Groups				T‐test	
		Patient		Control		t	*p* value
IMT (cm)	Range	0.06–0.31		0.06–0.15		4.642	<0.001*
	Mean ± SD	0.172 ± 0.070		0.105 ± 0.025			
Chi‐square		*N*	%	*N*	%	X^2^	*p* value
Focal thickness	No	23	60.53	22	84.62	4.292	0.038*
	Yes	15	39.47	4	15.38		
Focal thickness (location)	Bifurcation CCA	1	6.67	0	0.00	5.162	0.396
	Mid CCA	2	13.33	0	0.00		
	Distal CCA	3	20.00	3	75.00		
	Mid ICA	4	26.67	0	0.00		
	Proximal ICA	4	26.67	1	25.00		
	Proximal ECA	1	6.67	0	0.00		
Focal thickness (echogenicity)	Hyperechoic	10	66.67	3	75.00	0.101	0.750
	Hypoechoic	5	33.33	1	25.00		
Focal thickness (thickness, cm)	Range	0.24–0.46		0.32–0.41		0.171	0.866
	Mean ± SD	0.357 ± 0.074		0.350 ± 0.041			
Focal thickness (luminal diameter stenosis %)	Range	25–44.9		34–44.5		−0.627	0.539
	Mean ± SD	34.473 ± 7.119		36.875 ± 5.099			
Plaque	No	29	76.32	25	96.15	4.608	0.032*
	Yes	9	23.68	1	3.85		
Plaque (location)	Bifurcation CCA	1	11.11	0	0.00	1.111	0.774
	Distal CCA	4	44.44	1	100.00		
	Mid ICA	2	22.22	0	0.00		
	Proximal ICA	2	22.22	0	0.00		
Plaque (echogenicity)	Hyperechoic	7	77.78	1	100.00	0.278	0.598
	Hypoechoic	2	22.22	0	0.00		
Plaque (thickness, cm)	Range	0.55–0.69		0.57–0.57		0.966	0.362
	Mean ± SD	0.616 ± 0.045		0.570 ± 0.000			
Plaque (luminal diameter stenosis %)	Range	54–69.4		56.8–56.8		0.849	0.421
	Mean ± SD	61.111 ± 4.818		56.800 ± 0.000			

Abbreviations: CCA, common carotid artery; COPD, chronic obstructive pulmonary disease; ECA, external carotid artery; ICA, internal carotid artery; IMT, intima–media thickness.

* Statistically significant p < 0.05.

### 4.5 Laboratory results

Laboratory investigations revealed a significant elevation of serum inflammatory markers including CRP, fibrinogen, amyloid A, and IL6 in COPD patients than in controls (*p* value <0.001). There was also a significant increase of serum oxidative stress marker 8‐isoprostane in COPD patients than in controls (*p* value <0.001). Besides, there was a significant increase of serum vWF and UACR in COPD patients than in controls (*p* value <0.001) (see Supporting Information and Table 3).

**TABLE 3:** Laboratory investigations in COPD patients and control subjects

Laboratory investigations		Groups		T‐test	
		Patient	Control	t	*p* value
CRP (mg/l)	Range	1.9–82.3	0–10.3	3.924	<0.001*
	Mean ± SD	21.582 ± 20.700	5.500 ± 2.999		
Fibrinogen (g/l)	Range	2.2–25.7	1.8–5.9	5.295	<0.001*
	Mean ± SD	11.059 ± 6.769	3.931 ± 1.261		
Amyloid A (mg/l)	Range	6–77.8	2–14.9	4.085	<0.001*
	Mean ± SD	26.797 ± 22.370	8.692 ± 3.352		
IL6 (pg/ml)	Range	6.5–25	1–8	5.804	<0.001*
	Mean ± SD	11.295 ± 4.759	5.596 ± 1.843		
8‐isoprostane (pg/ml)	Range	80–250	40–100	6.207	<0.001*
	Mean ± SD	121.339 ± 38.716	70.500 ± 18.713		
vWF (ng/ml)	Range	8–30	5–10.5	5.755	<0.001*
	Mean ± SD	14.966 ± 6.561	7.431 ± 1.388		
UACR	Range	20–305	9.7–35	5.023	<0.001*
	Mean ± SD	112.842 ± 92.716	21.104 ± 7.081		

Abbreviations: COPD, chronic obstructive pulmonary disease; CRP, C‐reactive protein; UACR, urinary albumin creatinine ratio; vWF, von Willebrand factor.

* Statistically significant p < 0.05.

Serum CRP, fibrinogen, amyloid A, IL6, 8‐isoprostane, vWF, and UACR were positively correlated with mMRC, CAT score, risk of exacerbations, ABCD assessment tool grades, and the GOLD criteria but negatively correlated with FEV1/FVC ratio and FEV1 level (see Supporting Information and Tables 4 and 5).

**TABLE 4:** Correlation of the COPD severity assessment items with risk stratification of stroke, duplex findings, and laboratory investigations in COPD patients

Pearson correlation											
Variables		Spirometry post‐BD (FEV1/FVC)		FEV1 level (% predicted)		mMRC		CAT score		Risk of exacerbations	
		r	*p* value	r	*p* value	r	*p* value	r	*p* value	r	*p* value
Risk stratification of stroke	Stroke Riskometer™ (5‐year stroke risk%)	−0.681	<0.001*	−0.483	0.002*	0.326	0.046*	0.182	0.275	0.267	0.106
	Stroke Riskometer™ (10‐year stroke risk%)	−0.685	<0.001*	−0.475	0.003*	0.339	0.037*	0.203	0.221	0.279	0.090
	Framingham 10‐Year Risk Score (%)	−0.204	0.220	−0.689	<0.001*	0.251	0.129	0.224	0.177	0.144	0.387
	My Risk Stroke Calculator (points)	−0.530	0.001*	−0.069	0.681	0.253	0.125	0.223	0.179	0.245	0.139
	QRISK®3 10‐year risk calculator (%)	−0.531	0.001*	−0.605	<0.001*	0.336	0.039*	0.003	0.985	0.271	0.100
Duplex findings	IMT (cm)	−0.077	0.645	−0.607	<0.001*	0.051	0.759	0.113	0.498	0.011	0.946
	Focal thickness (thickness, cm)	−0.120	0.671	−0.360	0.188	0.005	0.987	0.028	0.921	0.014	0.962
	Focal thickness (luminal diameter stenosis %)	−0.066	0.817	−0.378	0.164	0.009	0.975	0.092	0.743	0.074	0.793
	Plaque (thickness, cm)	−0.079	0.840	−0.031	0.937	0.330	0.386	0.013	0.973	0.357	0.346
	Plaque (luminal diameter stenosis %)	−0.118	0.762	−0.011	0.978	0.347	0.361	0.032	0.935	0.341	0.369
Laboratory investigations	CRP (mg/l)	−0.600	<0.001*	−0.181	0.275	0.176	0.290	0.005	0.976	0.155	0.352
	Fibrinogen (g/l)	−0.181	0.277	−0.451	0.004*	0.165	0.322	0.154	0.354	0.107	0.522
	Amyloid A (mg/l)	−0.213	0.199	−0.389	0.016*	0.265	0.108	0.378	0.019*	0.057	0.735
	IL6(pg/ml)	−0.417	0.009*	−0.393	0.015*	0.115	0.492	0.097	0.562	0.198	0.233
	8‐isoprostane (pg/ml)	−0.229	0.167	−0.409	0.011*	0.250	0.130	0.049	0.769	0.014	0.934
	vWF (ng/ml)	−0.611	<0.001*	−0.284	0.084	0.055	0.741	0.023	0.891	0.209	0.208
	UACR	−0.452	0.004*	−0.362	0.025*	0.085	0.613	0.088	0.599	0.162	0.331

Abbreviations: CAT, COPD assessment test; COPD, chronic obstructive pulmonary disease; CRP, C‐reactive protein; FEV1, forced expiratory volume in 1 second; IMT, intima–media thickness; mMRC, modified Medical Research Council; post‐BD, post‐bronchodilator; UACR, urinary albumin creatinine ratio; vWF, von Willebrand factor.

* Statistically significant p < 0.05.

**TABLE 5:** Correlation of the ABCD assessment grades and GOLD criteria grades with risk stratification of stroke, duplex findings, and laboratory investigations in COPD patients

Spearman's rho correlation					
	Variables	ABCD assessment grades		GOLD criteria grades	
		r	*p* value	r	*p* value
Risk stratification of stroke	Stroke Riskometer™ (5‐year stroke risk%)	0.181	0.275	0.417	0.009*
	Stroke Riskometer™ (10‐year stroke risk%)	0.138	0.407	0.389	0.016*
	Framingham 10‐Year Risk Score (%)	0.034	0.840	0.622	<0.001*
	My Risk Stroke Calculator (points)	0.247	0.135	0.021	0.898
	QRISK®3 10‐year risk calculator (%)	0.068	0.684	0.472	0.003*
Duplex findings	IMT (cm)	0.133	0.427	0.591	<0.001*
	Focal thickness (thickness, cm)	0.361	0.187	0.039	0.889
	Focal thickness (luminal diameter stenosis %)	0.267	0.337	0.037	0.895
	Plaque (thickness, cm)	0.293	0.444	0.370	0.327
	Plaque (luminal diameter stenosis %)	0.329	0.388	0.369	0.329
	CRP (mg/l)	0.186	0.263	0.195	0.240
Laboratory investigations	Fibrinogen (g/l)	0.113	0.501	0.440	0.006*
	Amyloid A (mg/l)	0.095	0.569	0.352	0.030*
	IL6 (pg/ml)	0.087	0.603	0.316	0.053*
	8‐isoprostane (pg/ml)	0.055	0.745	0.320	0.050*
	vWF (ng/ml)	0.112	0.505	0.202	0.224
	UACR	0.081	0.630	0.250	0.129

Abbreviations: COPD, chronic obstructive pulmonary disease; CRP, C‐reactive protein; GOLD, Global Initiative for Chronic Obstructive Lung Disease; IMT, intima–media thickness; UACR, urinary albumin creatinine ratio; vWF, von Willebrand factor.

* Statistically significant p < 0.05.

## 5 DISCUSSION

Increasing evidence associates COPD with cerebrovascular disease in the elderly. A reduced pulmonary function is associated with an increased risk of stroke and subclinical events such as silent stroke, brain atrophy, or small vessel disease that have an impact on the quality of life.^22^

Patients diagnosed with COPD who were included in this study were linked to smoking. Patients had a mean smoking index of 47.5 ± 32.5 pack/year, 20 (52.63%) of them were smokers, 8 (21.05%) of them were ex‐smokers, and the remaining 10 (26.32%) were nonsmokers. This was in agreement with Wang et al. who stated that cigarette smoking was the most popular and the most important modifiable risk factor of COPD. The independent and positive relationship was basically established between cigarette smoking and COPD regardless that cigarette smoking was assessed with a categorical (smokers/ex‐smokers or nonsmokers) or continuous measure (number of cigarettes smoked).^23^

Regarding comorbidities recorded in this study, they showed nonsignificant positive correlation with Stroke Riskometer™ (5‐year stroke risk %), Stroke Riskometer™ (10‐year stroke risk %), Framingham 10‐Year Risk Score (%), and QRISK®3 10‐year risk calculator (%). In agreement with these results, Mannino et al. reported that although stroke risk factors are common in COPD patients, including hypertension, diabetes, and hypercholesterolaemia, stroke risk in COPD might not be wholly explained by the contribution of shared risk factors.^24^ O'Donnell et al. and Soderholm et al. also informed that major risk factors for stroke such as hypertension, current smoking, physical inactivity, diabetes mellitus, alcohol intake, and psychosocial stress and cardiac causes can also be seen in COPD patients, and thus, the link between COPD and stroke can be confounding.^25^, ^26^

Assessment of COPD severity in patients using refined ABCD assessment tool and GOLD criteria grades showed a significant reduction of spirometry‐assessed post‐brain diffusion (post‐BD) FEV1/FVC ratio and FEV1 level in COPD patients than in controls. There was a significant increase of mMRC, CAT score, and risk of exacerbations in COPD patients than in controls. According to ABCD assessment tool grades, 2 (5.26%) COPD patients were classified as Group A, 9 (23.68%) COPD patients were classified as Group B, 14 (36.84%) COPD patients were classified as Group C, and 13 (34.21%) COPD patients were classified as Group D. According to the GOLD criteria, 1 (2.63%) COPD patient was classified as Group 1, 7 (18.42%) COPD patients were classified as Group 2, 22 (57.89%) COPD patients were classified as Group 3, and 8 (21.05%) COPD patients were classified as Group 4. This was in line with Yawn who reported that the most commonly used COPD severity classification was the one proposed by the GOLD, which was based on a simple spirometry grading system, patient's symptom burden, and their history of exacerbations.^27^ This also was in agreement with Vogelmeier et al. who stated that the spirometric definition of obstruction using a fixed FEV1/FVC ratio of less than 0.70 has been consistent since the first GOLD guideline. The mMRC questionnaire and CAT were the two most widely used measures to assess symptoms of COPD. Symptom burden and risk of exacerbation were also further classified into GOLD Groups A through D, which is used to guide therapy.^28^

The current study showed a significant increase of each Stroke Riskometer™ (5‐year stroke risk), Stroke Riskometer™ (10‐year stroke risk), Framingham 10‐Year Risk Score, My Risk Stroke Calculator, and QRISK®3 10‐year risk calculator in COPD patients than in controls. According to the stroke risk screening tool, 4 (10.53%) patients were classified as having low risk, 14 (36.84%) patients were classified as having cautious risk, and 20 (52.63%) patients were classified as having high risk, with statistically highly significant differences than their matched controls. This matched with the results obtained by Hippisley et al., Nobel et al., Parmar et al., and Wolf et al. who showed that early and comprehensive risk identification would be critical to identify people at high risk for stroke. Therefore, comprehensive stroke risk screening instruments including the stroke risk screening tool, the Framingham 10‐Year Risk Score, The Stroke Riskometer™, Q Stroke, and the My Risk Stroke Calculator were needed to assess all possible stroke risks and potential at‐risk populations that would benefit early detection and stroke prevention planning.^29^, ^30^, ^31^, ^32^

Stroke Riskometer™ (5‐year stroke risk), Stroke Riskometer™ (10‐year stroke risk), Framingham 10‐Year Risk Score, My Risk Stroke Calculator, and QRISK®3 10‐year risk calculator were all directly correlated with mMRC, CAT score, risk of exacerbations, ABCD assessment tool grades, and the GOLD criteria but inversely correlated with FEV1/FVC ratio and FEV1 level. This was in accordance with Gulsvik et al. who concluded that interestingly decreased lung function expressed as decreased FEV1 and decreased FEV1/FVC ratio in COPD patients is correlated with an increased risk of stroke.^33^ In the same line, Portegies et al. reported that COPD exacerbations were also associated with an increased risk of stroke.^34^

In the present study, DTI has shown a significant reduction in fractional isotropy with a significant increase in MD in many areas including the SOFF, IOFF, arcuate fibers, corpus callosum, frontal subcortical tract, parietal subcortical tract, temporal subcortical tract, cingulum, corona radiata, internal capsule, cerebral peduncle, and corticospinal tract in both hemispheres in COPD patients than in controls. These results matched with those obtained from different studies carried by Zhang et al., Dodd et al., Yin et al., and Helmy et al. who concluded that patients with COPD had significantly reduced white matter microstructural integrity as shown by lower FA values and higher MD values in the white matter pathways in many regions that included the corpus callosum, cingulum, corona radiata, internal capsule, SOFF and IOFF, frontal subcortical tract, parietal subcortical tract, and temporal subcortical tract.^35^, ^36^, ^37^, ^38^ Besides, Evans et al. stated that they found that white matter diffusion measures and microstructural integrity impairment reflected by decreased FA and increased MD were associated with a higher risk of stroke, independent of white matter (WM) volume, intracranial volume, white matter lesion volume, and the presence of lacunar infarcts.^39^

T2 star revealed either normal findings in 18 (47.37%) COPD patients versus in 21 (80.77%) controls or microbleeds in 20 (52.63%) COPD patients versus in 5 (19.23%) controls. This was in agreement with a large population‐based study carried by Lahousse et al. and Maclay and MacNee, which showed that a higher prevalence of cerebral microbleeds was associated with COPD. It might be that patients with COPD are at risk for microbleeds due to comorbid processes, such as systemic inflammation without specific cognitive consequences or other clinical problems.^40^, ^41^ Besides, Greenberg et al. reported that COPD would preferentially lead to the development of deep or infratentorial microbleeds, which were thought to occur by arteriolosclerosis on the basis of hypertensive vasculopathy and lipohyalinosis. The risk on deep or infratentorial microbleeds increased with severity of airflow limitation, dyspnea symptoms, and exacerbation problems.^42^

MD of the SOFF, IOFF, arcuate fibers, corpus callosum, frontal subcortical tract, parietal subcortical tract, temporal subcortical tract, cingulum, corona radiata, internal capsule, cerebral peduncle, and corticospinal tract in both hemispheres was all directly correlated with mMRC, CAT score, risk of exacerbations, and the GOLD criteria but inversely correlated with FEV1/FVC ratio and FEV1 level. FA of the SOFF, IOFF, arcuate fibers, corpus callosum, frontal subcortical tract, parietal subcortical tract, temporal subcortical tract, cingulum, corona radiata, internal capsule, cerebral peduncle, and corticospinal tract in both hemispheres was all directly correlated with FEV1/FVC ratio and FEV1 level but inversely correlated with mMRC, CAT score, risk of exacerbations, ABCD assessment tool grades, and the GOLD criteria. These results were in line with Catherine et al. who found that lower lung function (FEV1 and FEV1/FVC) was associated with a deterioration in white matter macro‐ and microstructure evidenced by decreased FA and increased MD.^43^ Besides, Yin et al. found that relative to the comparison group, severe COPD patients showed the most extensive changes in WM integrity, including more decreased FA and increased MD, reflecting their correlation to symptom burden assessed by mMRC and CAT score.^37^ Besides, Helmy et al. reported a negative correlation between the number of exacerbations and the FA values of white matter tracts, which was not significant, whereas a significant positive correlation between the number of exacerbations and the MD values was seen in the majority of selected white matter tracts.^38^

In our study, color‐coded duplex ultrasonography showed a significant increase of IMT in COPD patients than in controls. This was in accordance with several studies investigating subclinical atherosclerosis accompanying COPD, which have reported that high carotid IMT (CIMT) was an important marker reflecting the early subclinical phase of atherosclerotic disease. In a review investigating the presence of subclinical atherosclerosis in COPD patients, 22 studies were examined, and it was found that CIMT was significantly higher in COPD patients than in the control groups in all of the studies in which CIMT was measured.^44^, ^45^

Focal thicknesses were present in 15 (39.47%) COPD patients with statistically significant differences than their matched controls, whereas plaques were present in nine (23.68%) COPD patients with statistically significant differences than their matched controls. Meanwhile, there were no statistically significant differences regarding location, echogenicity, thickness, and luminal diameter stenosis of either focal thickness or plaque in COPD patients than in controls. Recent studies carried by Iwamoto et al. and Lahousse et al. have shown that carotid arterial plaque burden and focal thickness were increased in patients with COPD and that these plaques were more prone to rupture, due to an increased lipid content, potentially leading to ischemic strokes.^46^, ^47^

IMT, thicknesses of both focal thicknesses and plaques, and luminal diameter stenosis of both focal thicknesses and plaques were positively correlated with mMRC, CAT score, risk of exacerbations, ABCD assessment tool grades, and the GOLD criteria but negatively correlated with FEV1/FVC ratio and FEV1 level. These results matched with those of the study carried by Kim et al., identifying a negative correlation between FEV1 level and CIMT measurements. A previous study made by Zureik et al. also reported a significant relationship between decreased FEV1 levels and endothelial dysfunction and vascular wall stiffness and the presence of atherosclerosis. Additionally, CIMT was found to be significantly associated with GOLD‐combined assessment groups as stated by Gulbas et al. Although previous studies reported no relationship between COPD exacerbations and CIMT, it has also been suggested by Golpe et al. that the increased systemic inflammatory response during exacerbations may contribute to the activation of an atherosclerotic process.^48^, ^49^, ^50^, ^51^ Ambrosino et al. found that the risk of carotid plaque development was directly correlated with GOLD‐combined assessment groups in COPD patients.^52^

Laboratory investigations revealed a significant elevation of serum inflammatory markers including CRP, fibrinogen, amyloid A, and IL6 in COPD patients than in controls. There was also a significant increase of serum oxidative stress marker 8‐isoprostane in COPD patients than in controls. Besides, there was a significant increase of serum vWF and UACR in COPD patients than in controls. These results matched with those of the studies conducted by Gan et al., Barnes et al., and Kazmierczak et al. who have shown, in addition to lung inflammation, that a state of chronic systemic inflammation was observed in COPD evidenced by increases in the serum levels of CRP, fibrinogen, serum amyloid A (SAA), and pro‐inflammatory cytokine including IL‐6 in COPD patients. Importantly, these markers of systemic inflammation were elevated even further during acute exacerbation. It was conceivable that the systemic inflammation and increased oxidative stress in COPD may independently increase stroke risk by directly promoting cerebral vascular dysfunction and thus vascular insufficiency.^19^, ^53^, ^54^ The study conducted by Gupta et al. indicated that there was a strong relationship of UACR in patients with COPD and the levels of microalbuminuria (MAB) increased as the severity of COPD increased due to hypoxia and endothelial dysfunction. As MAB was a marker for cardiovascular and cerebrovascular risks, patients with COPD can be routinely evaluated for the urine test of MAB specially those who were at an increased risk for cerebrovascular events.^55^ Besides, Bártholo et al. and Polosa et al. showed that vWF levels and relative activity have been found to be increased in COPD, which had an impact on platelet activation and a biomarker of endothelial dysfunction and inflammation in COPD.^56^, ^57^

Limitations of the study: It's worth mentioning that this study should be regarded in the perspective of a number of shortcomings. First, the restricted number of participants that has attributed to limited study duration, strict selection criteria, and the high costs of neuroimaging and assay kits. Second, the study could be more informative if we could do a follow‐up of our patients and evaluate the impact of proper management and prevention of cerebrovascular events. Therefore, future longitudinal prospective researches on a larger scale of participants are fundamental to identify whether patients at high risk for developing cerebrovascular insults as assessed by our research will be vulnerable for any insult or not.

## 6 CONCLUSION

COPD nowdays represents a major health problem worldwide, not only for patients but also for the community, so proper prevention and management of COPD and its exacerbation are now a vital target for a better quality of life. Systemic inflammation, oxidative stress, and shared risk factors involved in COPD pathogenesis should be targeted to prevent serious comorbidities such as cerebrovascular events. So, early screening of COPD patients with elevated inflammatory markers, oxidative burden, and poor lung functions for stroke risk becomes mandatory and valuable for both patients and the community.

## CONFLICT OF INTEREST

The researchers declared that they have no known competing financial interests or personal relations that could affect the work reported in this study.

## ETHICS STATEMENT

The study protocol was reviewed, was approved by the research ethics committee of the faculty of medicine, Tanta University (35 031/11/21), and has therefore been performed in agreement with the ethical standards laid down in the 1964 Declaration of Helsinki. A comprehensive clarification about the study was given by the researchers after which they provided consent for publication. All subjects enrolled in this study gave a written informed consent to publish the data contained within this study.

## AUTHORS' CONTRIBUTIONS

Marwa Badr conceptualized the study. Amira Elkholy, Sara Shoeib, Marwa Bahey, Esraa Mohamed and Alaa Reda have given inputs in study design. All authors shared in collecting the data. Marwa Badr analysed the data and wrote the first draft of the manuscript, and all co‐authors contributed in the critical review of data analysis and manuscript writing. Marwa Badr acts as the guarantor for this paper. All authors have read and approved the manuscript.

## COMPLIANCE WITH ETHICAL STANDARDS

All ethical standards were maintained. Any unexpected hazards that appeared during the research will be clarified to the subjects and the ethical committee on time. There were adequate measures to keep the privacy of participants and confidentiality of the data.

## Supporting information


**Table S1:** Demographic data in COPD patients and control subjects.
**Table S2:** COPD status assessment in COPD patients and control subjects.
**Table S3:** Risk stratification of stroke in COPD patients and control subjects.
**Table S4:** Correlation of comorbidity with risk stratification of stroke in COPD patients
**Table S5:** Correlation of the COPD severity assessment items with DTI in COPD patients
**Table S6:** Correlation of the ABCD assessment grades and GOLD criteria grades with DTI in COPD patients

## Data Availability

Regarding sharing materials, data will be available on request to the authors.
