# Supplementary material for: Assessment of incidence of cerebral vascular diseases and prediction of stroke risk in chronic obstructive pulmonary disease patients using multimodal biomarkers
Source: Clin Respir J. 2023 Jan 25;17(3):211–28. doi: 10.1111/crj.13587 (PMC9978912; doi:10.1111/crj.13587)
Supplement: Supplementary file 1 — Table S1: Demographic data in COPD patients and control subjects. Table S2: COPD status assessment in COPD patients and control subjects. Table S3: Risk stratification of stroke in COPD patients and control subjects. Table S4: Correlation of comorbidity with risk stratification of stroke in COPD patients Table S5: Correlation of the COPD severity assessment items with DTI in COPD patients Table S6: Correlation of the ABCD assessment grades and GOLD criteria grades with DTI in COPD patients [file CRJ-17-211-s001.docx]

Table 1: Demographic data in COPD patients and control subjects.

|  | | **Groups** | | | | | | **T-Test** | |
| --- | --- | --- | --- | --- | --- | --- | --- | --- | --- |
|  |  | **Patient** | | | **Control** | | | **t** | **P-value** |
| **Age (Years)** | **Range** | 44 | - | 67 | 45 | - | 65 | -3.386 | 0.001* |
|  | **Mean ±SD** | 55.5 | ± | 11.5 | 55 | ± | 10 |  |  |
| **Smoking index (pack/year)** | **Range** | 15 | - | 80 | 15 | - | 40 | -2.984 | 0.004* |
|  | **Mean ±SD** | 47.5 | ± | 32.5 | 27.5 | ± | 12.5 |  |  |
| **Chi-Square** | | **N** | | **%** | **N** | | **%** | **X^2^** | **P-value** |
| **Sex** | **Male** | 25 | | 65.79 | 17 | | 65.38 | 0.001 | 0.973 |
|  | **Female** | 13 | | 34.21 | 9 | | 34.62 |  |  |
| **Smoking** | **Non-Smoker** | 10 | | 26.32 | 16 | | 61.54 | 7.946 | 0.018* |
|  | **Smoker** | 20 | | 52.63 | 7 | | 26.92 |  |  |
|  | **Ex-Smoker** | 8 | | 21.05 | 3 | | 11.54 |  |  |
| **Co morbidity** | **Yes** | 19 | | 50.00 | 10 | | 38.46 | 0.829 | 0.362 |
|  | **No** | 19 | | 50.00 | 16 | | 61.54 |  |  |
| **Co morbidity** | **DM** | 7 | | 18.42 | 1 | | 3.85 | 2.998 | 0.083 |
|  | **HTN** | 18 | | 47.37 | 10 | | 38.46 | 0.498 | 0.481 |
|  | **IHD** | 1 | | 2.63 | 0 | | 0.00 | 0.695 | 0.404 |
|  | **Dyslipidemia** | 2 | | 5.26 | 0 | | 0.00 | 1.413 | 0.235 |
|  | **CKD** | 3 | | 7.89 | 1 | | 3.85 | 0.432 | 0.511 |

******* significant at p<0.05

Table 2: COPD status assessment in COPD patients and control subjects.

| **COPD status assessment** | | **Groups** | | | | | | **T-Test** | |
| --- | --- | --- | --- | --- | --- | --- | --- | --- | --- |
|  |  | **Patient** | | | **Control** | | | **t** | **P-value** |
| **Spirometry(FEV1/FVC)** | **Range** | 0.4 | - | 0.68 | 0.75 | - | 1.01 | -16.884 | <0.001* |
|  | **Mean ±SD** | 0.508 | ± | 0.089 | 0.881 | ± | 0.084 |  |  |
| **FEV1 level(% predicted)** | **Range** | 25 | - | 80 | 81 | - | 99 | -17.276 | <0.001* |
|  | **Mean ±SD** | 41.316 | ± | 13.804 | 90.654 | ± | 5.499 |  |  |
| **m MRC** | **Range** | 1 | - | 4 | 0 | - | 2 | 13.089 | <0.001* |
|  | **Mean ±SD** | 2.5 | ± | 1.5 | 0.731 | ± | 0.533 |  |  |
| **CAT score** | **Range** | 3 | - | 32 | 1 | - | 8 | 10.558 | <0.001* |
|  | **Mean ±SD** | 20.500 | ± | 7.270 | 5.038 | ± | 1.969 |  |  |
| **Risk of exacerbations(number/year)** | **Range** | 1 | - | 5 | - | - | - | - | - |
|  | **Mean ±SD** | 3.263 | ± | 1.223 | - | ± | - |  |  |
| **Chi-Square** | | **N** | | **%** | **N** | | **%** | **X^2^** | **P-value** |
| **ABCD assessment tool grades** | **Group A** | 2 | | 5.26 | - | | - | - | - |
|  | **Group B** | 9 | | 23.68 | - | | - |  |  |
|  | **Group C** | 14 | | 36.84 | - | | - |  |  |
|  | **Group D** | 13 | | 34.21 | - | | - |  |  |
| **GOLD criteria** | **Group 1** | 1 | | 2.63 | - | | - | - | - |
|  | **Group 2** | 7 | | 18.42 | - | | - |  |  |
|  | **Group 3** | 22 | | 57.89 | - | | - |  |  |
|  | **Group 4** | 8 | | 21.05 | - | | - |  |  |

******* significant at p<0.05

Table 3: Risk stratification of stroke in COPD patients and control subjects.

| **Risk stratification of stroke** | | **Groups** | | | | | | **T-Test** | |
| --- | --- | --- | --- | --- | --- | --- | --- | --- | --- |
|  |  | **Patient** | | | **Control** | | | **t** | **P-value** |
| **Stroke Riskometer TM(5 year stroke risk%)** | **Range** | 17 | - | 40 | 5 | - | 17.8 | 11.565 | <0.001* |
|  | **Mean ±SD** | 28.342 | ± | 6.373 | 12.396 | ± | 3.560 |  |  |
| **Stroke Riskometer TM(10 year stroke risk%)** | **Range** | 38 | - | 64.8 | 10 | - | 39 | 10.763 | <0.001* |
|  | **Mean ±SD** | 53.550 | ± | 8.675 | 28.119 | ± | 10.117 |  |  |
| **Framingham 10-Year Risk Score(%)** | **Range** | 10 | - | 33 | 3.9 | - | 11.8 | 10.670 | <0.001* |
|  | **Mean ±SD** | 22.068 | ± | 6.151 | 8.435 | ± | 2.551 |  |  |
| **My Risk Stroke Calculator(points)** | **Range** | 40 | - | 105 | 10 | - | 49 | 10.239 | <0.001* |
|  | **Mean ±SD** | 65.184 | ± | 18.682 | 23.923 | ± | 10.256 |  |  |
| **QRISK®3 10-year risk calculator(%)** | **Range** | 9.9 | - | 30.2 | 5.5 | - | 14.9 | 6.759 | <0.001* |
|  | **Mean ±SD** | 20.071 | ± | 6.441 | 11.012 | ± | 2.716 |  |  |
| **Chi-Square** | | **N** | | **%** | **N** | | **%** | **X^2^** | **P-value** |
| **Stroke Risk Screening Tool(points)** | **Low risk** | 4 | | 10.53 | 20 | | 76.92 | 32.769 | <0.001* |
|  | **Cautious** | 14 | | 36.84 | 6 | | 23.08 |  |  |
|  | **High risk** | 20 | | 52.63 | 0 | | 0.00 |  |  |
|  |  |  | |  |  | |  |  |  |

Table 4: Correlation of comorbidity with risk stratification of stroke in COPD patients

| **Variables** | **Co morbidity** | | | | | | **T-Test** | |
| --- | --- | --- | --- | --- | --- | --- | --- | --- |
|  | **Yes** | | | **No** | | |  |  |
|  | **Mean** | **±** | **SD** | **Mean** | **±** | **SD** | **t** | **P-value** |
| **Stroke RiskometerTM(5 year stroke risk%)** | 27.095 | ± | 6.659 | 29.589 | ± | 5.988 | -1.214 | 0.233 |
| **Stroke RiskometerTM(10 year stroke risk%)** | 51.979 | ± | 8.936 | 55.121 | ± | 8.344 | -1.120 | 0.270 |
| **Framingham 10-Year Risk Score(%)** | 20.647 | ± | 5.527 | 23.489 | ± | 6.554 | -1.445 | 0.157 |
| **My Risk Stroke Calculator(points)** | 66.789 | ± | 19.829 | 63.579 | ± | 17.855 | 0.524 | 0.603 |
| **QRISK®3 10-year risk calculator(%)** | 18.584 | ± | 5.980 | 21.558 | ± | 6.697 | -1.444 | 0.157 |

******* significant at p<0.05

Table 5: Correlation of the COPD severity assessment items with DTI in COPD patients

| **Pearson Correlation** | | | | | | | | | | | |
| --- | --- | --- | --- | --- | --- | --- | --- | --- | --- | --- | --- |
| **Variable(DTI)** | | **Spirometry** **post-BD**  **(FEV1/FVC)** | | **Spirometry FEV1**  **level(%predicted)** | | **m MRC** | | **CAT**  **score** | | **Risk of**  **exacerbations** | |
|  |  | **r** | **P-value** | **r** | **P-value** | **r** | **P-value** | **r** | **P-value** | **r** | **P-value** |
| **SOFF(FA)** | **RT** | 0.304 | 0.063 | 0.439 | 0.006* | -0.079 | 0.635 | -0.012 | 0.942 | -0.106 | 0.528 |
|  | **LT** | 0.310 | 0.058 | 0.419 | 0.009* | -0.035 | 0.835 | -0.021 | 0.901 | -0.133 | 0.426 |
| **SOFF(MD)** | **RT** | -0.652 | <0.001* | -0.157 | 0.347 | 0.177 | 0.288 | 0.081 | 0.629 | 0.229 | 0.166 |
|  | **LT** | -0.599 | <0.001* | -0.185 | 0.266 | 0.091 | 0.586 | 0.039 | 0.814 | 0.307 | 0.061 |
| **IOFF(FA)** | **RT** | 0.310 | 0.058 | 0.637 | <0.001* | -0.189 | 0.256 | -0.081 | 0.630 | -0.055 | 0.744 |
|  | **LT** | 0.263 | 0.111 | 0.625 | <0.001* | -0.197 | 0.236 | -0.078 | 0.640 | -0.023 | 0.891 |
| **IOFF(MD)** | **RT** | -0.522 | 0.001* | -0.362 | 0.026* | 0.092 | 0.583 | 0.116 | 0.488 | 0.390 | 0.016* |
|  | **LT** | -0.437 | 0.006* | -0.463 | 0.003* | 0.070 | 0.677 | 0.023 | 0.891 | 0.113 | 0.498 |
| **arcuate F(FA)** | **RT** | 0.318 | 0.052* | 0.417 | 0.009* | -0.326 | 0.046* | -0.267 | 0.105 | -0.421 | 0.008* |
|  | **LT** | 0.445 | 0.005* | 0.483 | 0.002* | -0.275 | 0.095 | -0.196 | 0.239 | -0.377 | 0.019* |
| **arcuate F(MD)** | **RT** | -0.425 | 0.008* | -0.083 | 0.622 | 0.046 | 0.784 | 0.169 | 0.310 | 0.286 | 0.082 |
|  | **LT** | -0.055 | 0.742 | -0.203 | 0.222 | 0.288 | 0.079 | 0.037 | 0.827 | 0.197 | 0.235 |
| **CC(FA)** |  | 0.615 | <0.001* | 0.416 | 0.009* | -0.324 | 0.047* | -0.316 | 0.053* | -0.327 | 0.045* |
| **CC(MD)** |  | -0.177 | 0.288 | -0.299 | 0.068 | 0.028 | 0.866 | 0.249 | 0.132 | 0.044 | 0.792 |
| **frontal subcortical tract(FA)** | **RT** | 0.351 | 0.031* | 0.771 | <0.001* | -0.408 | 0.011* | -0.142 | 0.394 | -0.277 | 0.092 |
|  | **LT** | 0.197 | 0.235 | 0.760 | <0.001* | -0.350 | 0.031* | -0.060 | 0.721 | -0.289 | 0.079 |
| **frontal subcortical tract(MD)** | **RT** | -0.566 | <0.001* | -0.493 | 0.002* | 0.409 | 0.011* | 0.222 | 0.181 | 0.420 | 0.009* |
|  | **LT** | -0.559 | <0.001* | -0.552 | <0.001* | 0.367 | 0.024* | 0.201 | 0.227 | 0.397 | 0.014* |
| **parietal subcortical tract(FA)** | **RT** | 0.478 | 0.002* | 0.694 | <0.001* | -0.412 | 0.010* | -0.147 | 0.380 | -0.365 | 0.024* |
|  | **LT** | 0.013 | 0.937 | 0.634 | <0.001* | -0.342 | 0.036* | -0.038 | 0.819 | -0.230 | 0.164 |
| **parietal subcortical tract(MD)** | **RT** | -0.703 | <0.001* | -0.466 | 0.003* | 0.309 | 0.059 | 0.180 | 0.280 | 0.279 | 0.090 |
|  | **LT** | -0.671 | <0.001* | -0.548 | <0.001* | 0.346 | 0.033* | 0.197 | 0.235 | 0.340 | 0.037* |
| **temporal subcortical tract(FA)** | **RT** | 0.027 | 0.871 | 0.626 | <0.001* | 0.118 | 0.481 | 0.167 | 0.317 | -0.032 | 0.848 |
|  | **LT** | 0.381 | 0.018* | 0.302 | 0.065 | 0.147 | 0.379 | -0.047 | 0.780 | -0.123 | 0.462 |
| **temporal subcortical tract(MD)** | **RT** | -0.126 | 0.451 | -0.713 | <0.001* | 0.314 | 0.055 | 0.233 | 0.159 | 0.166 | 0.318 |
|  | **LT** | -0.090 | 0.592 | -0.561 | <0.001* | 0.373 | 0.021* | 0.096 | 0.568 | 0.303 | 0.065 |
| **cingulum(FA)** | **RT** | 0.177 | 0.287 | 0.768 | <0.001* | -0.353 | 0.030* | -0.149 | 0.371 | -0.266 | 0.107 |
|  | **Lt** | 0.285 | 0.083 | 0.738 | <0.001* | -0.377 | 0.020* | -0.203 | 0.222 | -0.366 | 0.024* |
| **cingulum(MD)** | **RT** | -0.311 | 0.057 | -0.677 | <0.001* | 0.420 | 0.009* | 0.111 | 0.507 | 0.279 | 0.090 |
|  | **LT** | -0.726 | <0.001* | -0.304 | 0.063 | 0.316 | 0.053* | 0.267 | 0.104 | 0.333 | 0.041* |
| **corona radiata(FA)** | **RT** | 0.034 | 0.839 | 0.759 | <0.001* | -0.161 | 0.333 | -0.057 | 0.733 | -0.095 | 0.569 |
|  | **LT** | 0.184 | 0.270 | 0.810 | <0.001* | -0.317 | 0.052* | -0.154 | 0.355 | -0.152 | 0.362 |
| **corona radiata(MD)** | **RT** | -0.290 | 0.077 | -0.625 | <0.001* | 0.399 | 0.013* | 0.278 | 0.091 | 0.217 | 0.191 |
|  | **LT** | -0.691 | <0.001* | -0.396 | 0.014* | 0.423 | 0.008* | 0.259 | 0.116 | 0.414 | 0.010* |
| **internal capsule(FA)** | **RT** | 0.342 | 0.035* | 0.686 | <0.001* | -0.276 | 0.093 | -0.064 | 0.701 | -0.243 | 0.141 |
|  | **LT** | 0.440 | 0.006* | 0.686 | <0.001* | -0.406 | 0.011* | -0.272 | 0.099 | -0.323 | 0.048* |
| **internal capsule(MD)** | **RT** | -0.489 | 0.002* | -0.673 | <0.001* | 0.362 | 0.026* | 0.151 | 0.365 | 0.337 | 0.038* |
|  | **LT** | -0.612 | <0.001* | -0.208 | 0.210 | 0.218 | 0.189 | 0.032 | 0.849 | 0.136 | 0.415 |
| **cerebral peduncle(FA)** | **RT** | 0.366 | 0.024* | 0.747 | <0.001* | -0.213 | 0.200 | -0.301 | 0.067 | -0.216 | 0.192 |
|  | **LT** | 0.158 | 0.345 | 0.134 | 0.424 | -0.071 | 0.673 | -0.309 | 0.059 | -0.312 | 0.056 |
| **cerebral peduncle(MD)** | **RT** | -0.717 | <0.001* | -0.378 | 0.019* | 0.276 | 0.093 | 0.211 | 0.204 | 0.303 | 0.064 |
|  | **LT** | -0.636 | <0.001* | -0.162 | 0.331 | 0.164 | 0.326 | 0.316 | 0.054* | 0.144 | 0.387 |
| **cortico-spinal tract(FA)** | **RT** | 0.083 | 0.620 | 0.754 | <0.001* | -0.376 | 0.020* | -0.197 | 0.235 | -0.191 | 0.250 |
|  | **LT** | 0.493 | 0.002* | 0.654 | <0.001* | -0.418 | 0.009* | -0.285 | 0.083 | -0.354 | 0.029* |
| **cortico-spinal tract(MD)** | **RT** | -0.402 | 0.012* | -0.522 | 0.001* | 0.320 | 0.050* | 0.009 | 0.957 | 0.315 | 0.054* |
|  | **LT** | -0.492 | 0.002* | -0.622 | <0.001* | 0.461 | 0.004* | 0.232 | 0.160 | 0.348 | 0.032* |

******* significant at p<0.05

Table 6: Correlation of the ABCD assessment grades and GOLD criteria grades with DTI in COPD patients

| **Spearman's rho correlation** | | | | | |
| --- | --- | --- | --- | --- | --- |
| **Variable(DTI)** | | **ADCD assessment grades** | | **GOLD criteria** | |
|  |  | **r** | **P-value** | **r** | **P-value** |
| **SOFF(FA)** | **RT** | **-0.002** | **0.991** | **-0.432** | **0.007*** |
|  | **LT** | **-0.016** | **0.926** | **-0.434** | **0.007*** |
| **SOFF(MD)** | **RT** | **0.048** | **0.777** | **0.083** | **0.621** |
|  | **LT** | **0.026** | **0.879** | **0.169** | **0.310** |
| **IOFF(FA)** | **RT** | **-0.044** | **0.792** | **-0.585** | **<0.001*** |
|  | **LT** | **-0.081** | **0.630** | **-0.563** | **<0.001*** |
| **IOFF(MD)** | **RT** | **0.310** | **0.059** | **0.371** | **0.022*** |
|  | **LT** | **0.089** | **0.596** | **0.464** | **0.003*** |
| **arcuate F(FA)** | **RT** | **-0.207** | **0.212** | **-0.350** | **0.031*** |
|  | **LT** | **-0.018** | **0.912** | **-0.523** | **0.001*** |
| **arcuate F(MD)** | **RT** | **0.221** | **0.182** | **0.342** | **0.036*** |
|  | **LT** | **0.096** | **0.568** | **0.452** | **0.004*** |
| **CC(FA)** |  | **-0.173** | **0.299** | **-0.384** | **0.017*** |
| **CC(MD)** |  | **0.001** | **0.993** | **0.613** | **<0.001*** |
| **frontal subcortical tract(FA)** | **RT** | **-0.076** | **0.648** | **-0.627** | **<0.001*** |
|  | **LT** | **-0.065** | **0.698** | **-0.602** | **<0.001*** |
| **frontal subcortical tract(MD)** | **RT** | **0.081** | **0.627** | **0.408** | **0.011*** |
|  | **LT** | **0.114** | **0.497** | **0.507** | **0.001*** |
| **parietal subcortical tract(FA)** | **RT** | **-0.101** | **0.548** | **-0.505** | **0.001*** |
|  | **LT** | **-0.004** | **0.981** | **-0.580** | **<0.001*** |
| **parietal subcortical tract(MD)** | **RT** | **0.168** | **0.313** | **0.402** | **0.012*** |
|  | **LT** | **0.199** | **0.230** | **0.410** | **0.011*** |
| **temporal subcortical tract(FA)** | **RT** | **-0.072** | **0.668** | **-0.554** | **<0.001*** |
|  | **LT** | **-0.246** | **0.137** | **-0.196** | **0.239** |
| **temporal subcortical tract(MD)** | **RT** | **0.045** | **0.787** | **0.592** | **<0.001*** |
|  | **LT** | **0.012** | **0.943** | **0.624** | **<0.001*** |
| **cingulum(FA)** | **RT** | **-0.108** | **0.518** | **-0.575** | **<0.001*** |
|  | **Lt** | **-0.183** | **0.272** | **-0.525** | **0.001*** |
| **cingulum(MD)** | **RT** | **0.125** | **0.454** | **0.633** | **<0.001*** |
|  | **LT** | **0.156** | **0.350** | **0.384** | **0.017*** |
| **corona radiata(FA)** | **RT** | **-0.037** | **0.827** | **-0.728** | **<0.001*** |
|  | **LT** | **-0.023** | **0.890** | **-0.723** | **<0.001*** |
| **corona radiata(MD)** | **RT** | **0.071** | **0.670** | **0.620** | **<0.001*** |
|  | **LT** | **0.164** | **0.326** | **0.363** | **0.025*** |
| **internal capsule(FA)** | **RT** | **-0.091** | **0.585** | **-0.429** | **0.007*** |
|  | **LT** | **-0.192** | **0.248** | **-0.604** | **<0.001*** |
| **internal capsule(MD)** | **RT** | **0.111** | **0.508** | **0.574** | **<0.001*** |
|  | **LT** | **0.121** | **0.469** | **0.406** | **0.011*** |
| **cerebral peduncle(FA)** | **RT** | **-0.093** | **0.580** | **-0.654** | **<0.001*** |
|  | **LT** | **-0.079** | **0.635** | **-0.139** | **0.405** |
| **cerebral peduncle(MD)** | **RT** | **0.146** | **0.382** | **0.415** | **0.010*** |
|  | **LT** | **0.163** | **0.328** | **0.409** | **0.011*** |
| **cortico-spinal tract(FA)** | **RT** | **-0.038** | **0.819** | **-0.653** | **<0.001*** |
|  | **LT** | **-0.136** | **0.415** | **-0.488** | **0.002*** |
| **cortico-spinal tract(MD)** | **RT** | **0.134** | **0.424** | **0.587** | **<0.001*** |
|  | **LT** | **0.161** | **0.335** | **0.518** | **0.001*** |

******* significant at p<0.05
